# A knowledge-based framework for robust segmentation of high-resolution impedance manometry catheters in video-fluoroscopy images

**DOI:** 10.1007/s11548-026-03593-4

**Published:** 2026-04-04

**Authors:** Manuel Maria Loureiro da Rocha, Dionne S.Brandsma, Lisette van der Molen, Maarten J. A. van Alphen, Michiel W. M. van den Brekel, Françoise J. Siepel

**Affiliations:** 1 Robotics and Mechatronics Group, University of Twente, Drienerlolaan 5, 7522 NB Enschede, The Netherlands; 2 Head and Neck Oncology, Netherlands Cancer Institute, Plesmanlaan 121, 1066 CX Amsterdam, The Netherlands

**Keywords:** Manometer, Swallowing videos, Head and neck cancer, Automatic segmentation, Oropharyngeal dysphagia

## Abstract

**Purpose:**

Simultaneous high-resolution impedance manometry (HRIM) and videofluoroscoic swallow studies (VFSS) can address important limitations of VFSS. However, clinicians must analyze each modality independently, doubling workload and leaving the challenge of manometric region delineation unresolved. We hypothesize that spatially registering HRIM and VFSS would allow manometric regions to be defined directly on VFSS frames, reducing clinician burden. Achieving this requires reliable detection of the HRIM catheter in VFSS images.

**Methods:**

We introduce a template-free, knowledge-based algorithm that automatically localizes the catheter centreline in VFSS frames. The method identifies the main visible portion of the catheter in each frame, and then recursively adds catheter segments based on proximity and directional alignment. This approach defines a region-of-interest containing the individual HRIM sensors. The algorithm was validated on two datasets comprising frames from 122 single-swallow VFSS videos of head and neck cancer patients.

**Results:**

The segmentation module achieved 93.8% precision, 83.8% recall, and an F1-score of 88.5% for a 1.77 mm tolerance.

**Conclusion:**

The framework demonstrated robust performance across diverse anatomies and imaging conditions, outperforming existing knowledge-based methods. By relying on geometric and directional priors rather than pixel intensities, it delivers consistent, interpretable predictions without requiring large annotated datasets. This algorithm lays the groundwork for manometric region delineation directly on imaging data, and could likely be extended to other clinical applications involving thin radiopaque structures, provided that the pre-processing pipeline is adjusted and the hyperparameters are appropriately fine-tuned.

## Introduction

### Head and neck cancer

Head and neck cancer (HNC) is the sixth most common type of cancer worldwide, according to GLOBOCAN 2022 [1]. It includes malignancies of the upper-aerodigestive tract, namely the oral cavity, nasopharynx, (oro)pharynx and larynx [2]. Due to the location of the affected structures and the side effects of treatment modalities [3], HNC patients often develop oropharyngeal dysphagia (OD), i.e., swallowing impairment. OD severely reduces the quality of life of patients, causing malnutrition, dehydration, aspiration-induced pneumonia or even death [1, 4].

A gold standard diagnostic tool of OD is the videofluoroscopic swallow study (VFSS) [5]. In VFSS, patients ingest boluses of different volumes and consistencies mixed with a contrast agent, while fluoroscopy videos capture each individual swallow [6]. VFSS provides a detailed, dynamic, and real-time assessment of the swallowing process, allowing the visualization of all swallowing phases (i.e., oral preparatory, oral propulsive, pharyngeal, and esophageal) and the identification of specific dysfunctions (e.g., oropharyngeal residue, laryngeal penetration/aspiration), which makes it a crucial diagnostic tool in dysphagia management [7]. Despite its clinical value, VFSS analysis still relies heavily on clinician interpretation, making it susceptible to inter-rater variability and bias [8, 9].

High-Resolution Impedance Manometry (HRIM) provides a quantitative swallow assessment by measuring the pressure and impedance variations during swallowing [10, 11]. Key swallow metrics are then derived from the pressure/impedance signals [12, 13], such as regional contractile integrals, UES relaxation time or bolus presence time, using validated semi-automated programs (e.g., SwallowGateway (Australia)) [14, 15]. However, HRIM analysis requires manual steps from the clinicians, including main-swallow identification and manometric region delineation (velopharynx, mesopharynx, hypopharynx and upper esophageal sphincter) [12]. These steps are crucial to ensure the accuracy of the semi-automated analysis programs [14], as small changes in region delineation can substantially alter key swallow quality indicators, potentially resulting in inaccurate diagnosis and ill-guided recovery plans. In HNC patients this task is even more challenging and less reliable, as treatment-related anatomical changes often result in reduced pharyngeal pressures [8, 11] and distorted impedance patterns. As a result, the established guidelines for manometric region delineation in HRIM plots [12], many of which rely on identifying clear pressure signatures, are often difficult or impossible to apply.

Although simultaneous HRIM-VFSS helps address important limitations of VFSS, combining both modalities still requires clinicians to analyze each study independently, effectively doubling the workload, and leaving the fundamental challenges of HRIM, particularly the difficulty of accurate manometric region delineation, unresolved [16–18]. To overcome these issues, we hypothesize that achieving robust spatial registration between VFSS and HRIM would allow manometric region delineation to be performed directly on the VFSS frames, using either manual or automated methods, rather than relying solely on the HRIM plots. Enabling such registration first requires the automatic detection of the manometric catheter within the VFSS frames.

### Automatic segmentation in fluoroscopy imaging

In recent years, deep-learning-based methodologies have been increasingly applied to different segmentation tasks in fluoroscopy imaging. While Wang et al. [19] used an HRNet for catheter segmentation, U-Net models have remained the preferred choice across a variety of medical procedures, including liver catheterization [20], angiography [21–23], chest x-rays [24] and endoscopic interventions [25]. In VFSS, segmentation-oriented deep-learning architectures, such as U-Nets and Mask R-CNN, have been applied to segment and track the bolus during swallows [26, 27].

Despite driving major advances in automating medical data analysis, deep-learning models typically require large, diverse, and high-quality annotated datasets to perform well and enable real-world deployment [28]. However, in many clinical settings such datasets are difficult to obtain, limiting the practical impact of deep-learning-based solutions. In contrast, knowledge-based approaches offer an effective, low-data alternative to deep-learning approaches. By leveraging domain-specific information, they can achieve robust performances in segmentation tasks while requiring much less annotated data.

In the literature, several knowledge-based segmentation approaches have been proposed for extracting thin structures across a range of fluoroscopy scenarios. Wu et al. [29] introduced a patch-growing catheter segmentation method using Speeded Up Robust Features (SURF) combined with Markov Random Fields (MRF) and Kalman filters for cardiac procedures. Building on related principles, Chang et al. [30], Wang et al. [31], and Hoffmann et al. [32] focused on detecting and tracking catheters/guidewires in endovascular scenes, employing a range of strategies from probabilistic frameworks to graph-based analysis. More recently, Omisore et al. [33] developed a promising knowledge-based segmentation algorithm to detect guidewires in cardiac robotic catheterization. Together, these methods illustrate the effectiveness of knowledge-based strategies for thin-structure segmentation in simple imaging environments. However, VFSS presents a particularly challenging setting for such approaches: the videos are characterized by fast dynamics, such as rapid bolus flow, and contain complex backgrounds involving overlapping anatomical structures. Algorithms aiming to detect features like catheters must therefore handle partial occlusions and reliably separate the target from a highly cluttered background. Consequently, despite their promising results, existing knowledge-based techniques are not easily deployable to VFSS segmentation tasks, as they were primarily designed and validated under conditions with limited motion and relatively clean backgrounds.

Recent pilot frameworks by Jell et al. [34] and Geiger et al. [35] introduced two-stage template-matching approaches for localizing the HRIM catheter and detecting its individual sensors in VFSS videos. These methods demonstrated technical feasibility and highlighted the clinical potential of integrating HRIM and VFSS into a single interface to reduce clinician workload. However, because both frameworks rely on template matching, their generalization is limited across different catheter models, restricting broader clinical applicability.

To address all these limitations, we introduce a template-free, knowledge-based segmentation algorithm that automatically localizes the centreline of the HRIM catheter in VFSS images, under challenging conditions such as variable lighting, catheter curvature, and occlusions. The extracted centerline defines a region-of-interest (ROI) within the VFSS sequence in which the individual HRIM sensors are expected to lie. This ROI provides the foundation for a subsequent algorithm capable of detecting and identifying each sensor, thereby enabling complete spatial registration between HRIM and VFSS.

## Materials and methods

### Data collection protocol

This study included retrospective post-treatment data from 16 HNC patients. The capture of simultaneous VFSS and HRIM was conducted by a team of trained speech-language pathologists at the Netherlands Cancer Institute (NKI) (Amsterdam, the Netherlands). Patients were seated in a lateral up-right position ensuring the visibility of the HRIM catheter and all relevant anatomical structures in the fluoroscopy videos. VFSS videos were acquired at 25 frames-per-second (fps) using the CombiDiagnost R90$^\mathtt{{TM}}$ (Philips®, Amsterdam, the Netherlands) by experienced speech-language pathologists and radiology technicians. The Solar GI HRIM Solid State Trolley System (SN 18710476, Laborie®, Enschede, the Netherlands) combined with the 3.3 mm solid-state catheter Unisensor HRM K103659-E-118-D (Laborie®, Enschede, the Netherlands) acquired pressure and impedance data at 20 Hz. The catheter included 36 pressure and 16 impedance sensors.

Before each VFSS examination, the solid-state manometer was transnasally inserted by a speech-language pathologist following the procedural recommendation of Laborie®, and positioned so that its distal sensors extended beyond the upper esophageal sphincter. During assessment, patients were given and asked to swallow boluses of different amounts and consistences, prepared following the International Dysphagia Diet Standardization Initiative (IDDSI) [36]. To achieve a diversified and representative characterization of the swallowing function, the protocol aimed to obtain at least one, and ideally two, swallows per patient for each volume (5 mL and 10 mL), and for three consistency categories: thin or slightly thick liquids (IDDSI 0–1), moderately or extremely thick liquids (IDDSI 3–4), and regular solids (IDDSI 7; contrast coated crackers and gingerbread). To ensure a progressive characterization of swallowing, boluses were administered in increasing order of thickness and volume (e.g., IDDSI 0 at 5 mL followed by IDDSI 0 at 10 mL), allowing adjustments where clinically necessary, such as when patients were unable to safely swallow specific consistencies. In some cases, dry swallows were also recorded to support the clinical evaluation. The contrast residue used was Omnipaque$^\mathtt{{TM}}$ 300 mg/ml mixed with the Standardized Bolus Medium (SBMkit) product (Trisco, Pty. Ltd., Australia) containing the saline concentrate required for impedance measurements according to Omari et al. [12].

### Datasets

**Fig. 1:**
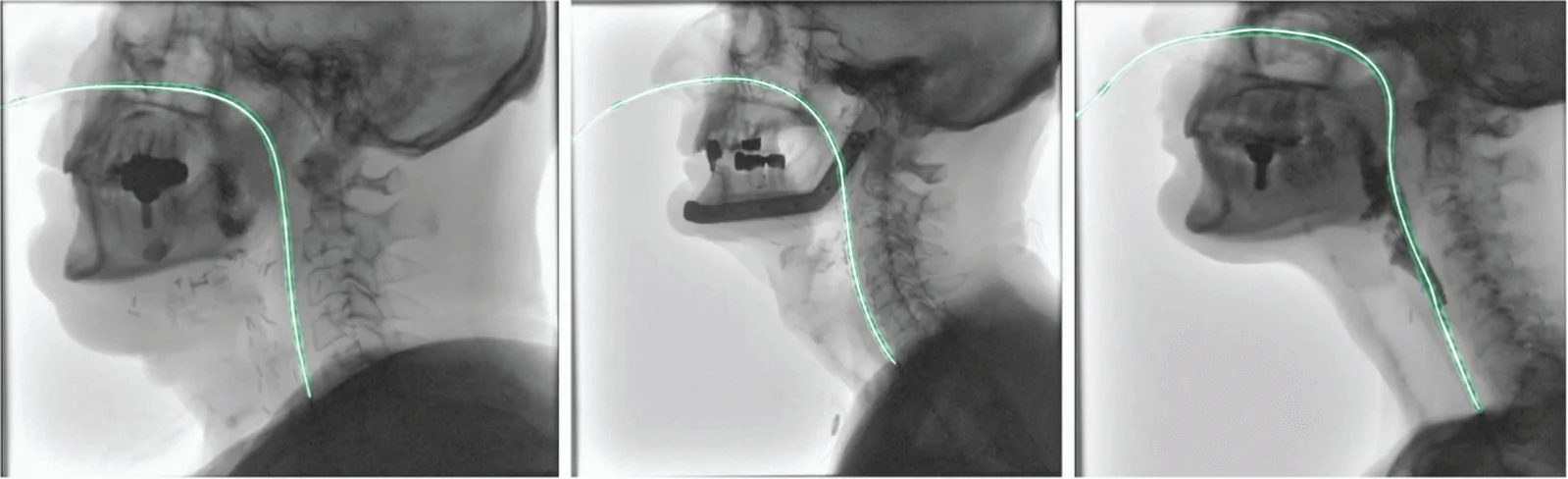
Example VFSS images with annotations. The ground-truth catheter masks (green) cover the entire visible catheter up to the shoulder region, with the corresponding centrelines shown in white. For visualization purposes, the catheter centrelines were enlarged

This study included 122 single-swallow VFSS videos collected under NKI-AVL Institutional Review Board’s approval (IRBd21-210, IRBd23-322). From these recordings, two datasets were constructed. The first, the *Random Samples Dataset*, contained 976 frames sampled uniformly at random from the full set of videos, and was used to evaluate the algorithm’s overall robustness across a wide range of anatomies and imaging conditions. The second, the *Full Videos Dataset*, included 1631 frames extracted from 7 complete swallow sequences, providing access to the algorithm’s performance in continuous, dynamic scenes, with guaranteed bolus-induced occlusions. For both datasets, catheter masks were manually delineated in Labelbox © (CA, USA), ensuring each mask captured the entire visible catheter up to the shoulder region, shown in Fig. 1.

Pixel-to-millimetre scales were obtained for each swallow video by manually identifying the start and end of a sensor in the first frame. Using the known physical spacing of the catheter model (1 cm), pixel distances were converted to metric units.

### Pre-processing

**Fig. 2:**
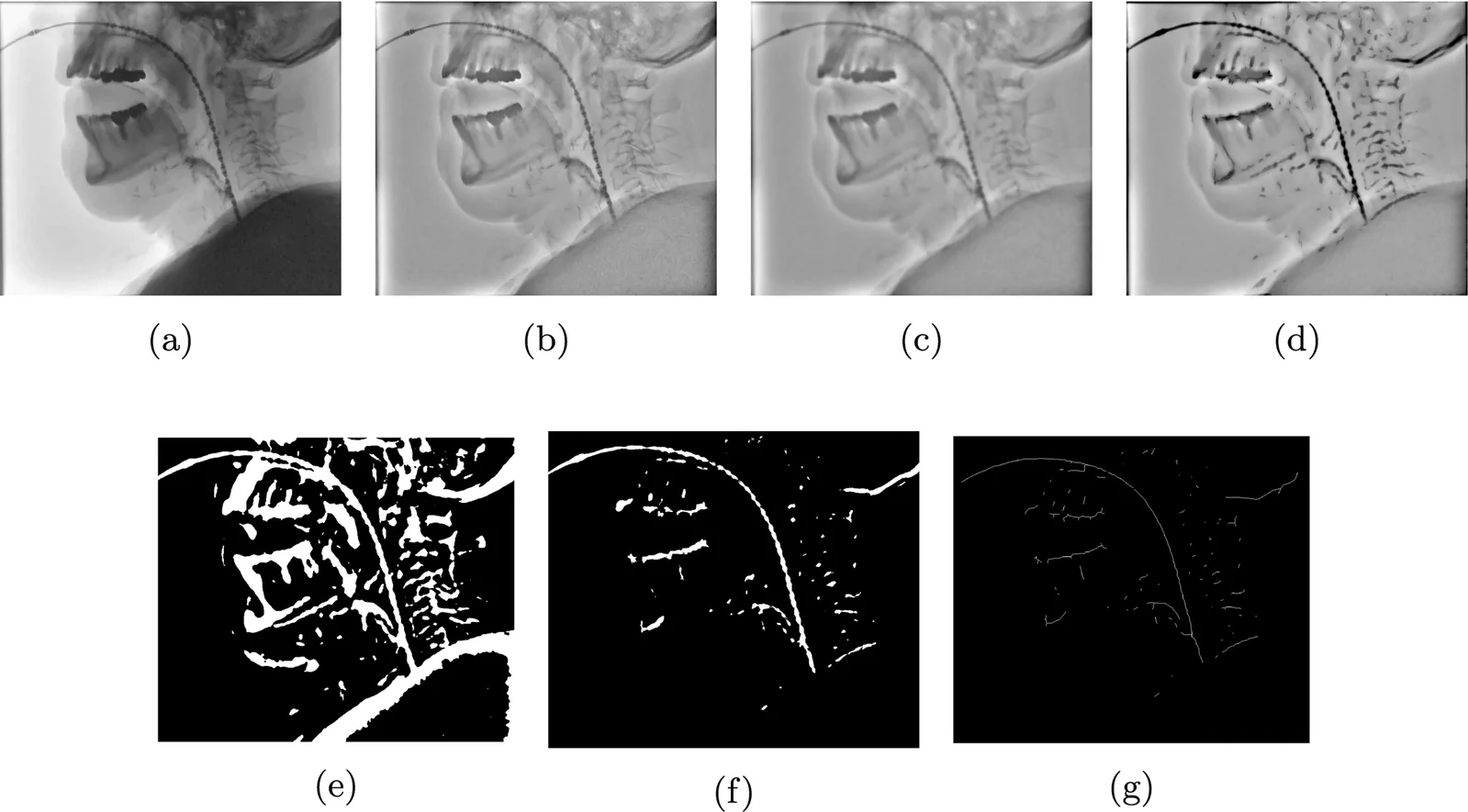
Pre-processing pipeline to limit the catheter’s search region. **a** Original VFSS frame, **b** high-pass Gaussian filter, **c** Gaussian smoothing, **d** bottom-hat subtraction result, **e** initial response of the adaptive thresholding algorithm, **f** final catheter search region, and **g** its skeletonized version

Pre-processing techniques constrain the search region for HRIM catheter localization. As shown in Fig. 2a, the catheter typically appears as a dark structure with sharp intensity transitions. To enhance its boundaries, we first apply a high-pass Gaussian filter (cf. Fig. 2b), followed by a Gaussian smoothing operation to reduce noise (cf. Fig. 2c). A bottom-hat morphological operator with cross-shaped and diagonal structuring elements then highlights the dark catheter regions. The result is thresholded and used to suppress dark areas in the original image, producing a distinct dark structure against a uniform background output (cf. Fig. 2d).

The result is refined using an adaptive area-based thresholding algorithm, that iteratively reduces the intensity threshold until a minimum proportion of dark pixels is detected. This method attends to the consistent sensor size while accounting for the variable illumination or contrast. Morphological closing operations further constrain the search region for catheter localization (cf. Fig. 2f).

### Catheter segmentation

The catheter segmentation algorithm operates on a skeletonized representation of the pre-processed search region, which often contains multiple irrelevant connected components (see Fig. 2g). To address this, we introduce a recursive approach that filters components based on geometric continuity, exploiting the smooth curvature and gradual transitions characteristic of HRIM catheters. The algorithm consists of three stages: main component selection, component pruning, and component propagation.

#### Main component selection and characterization

**Fig. 3:**
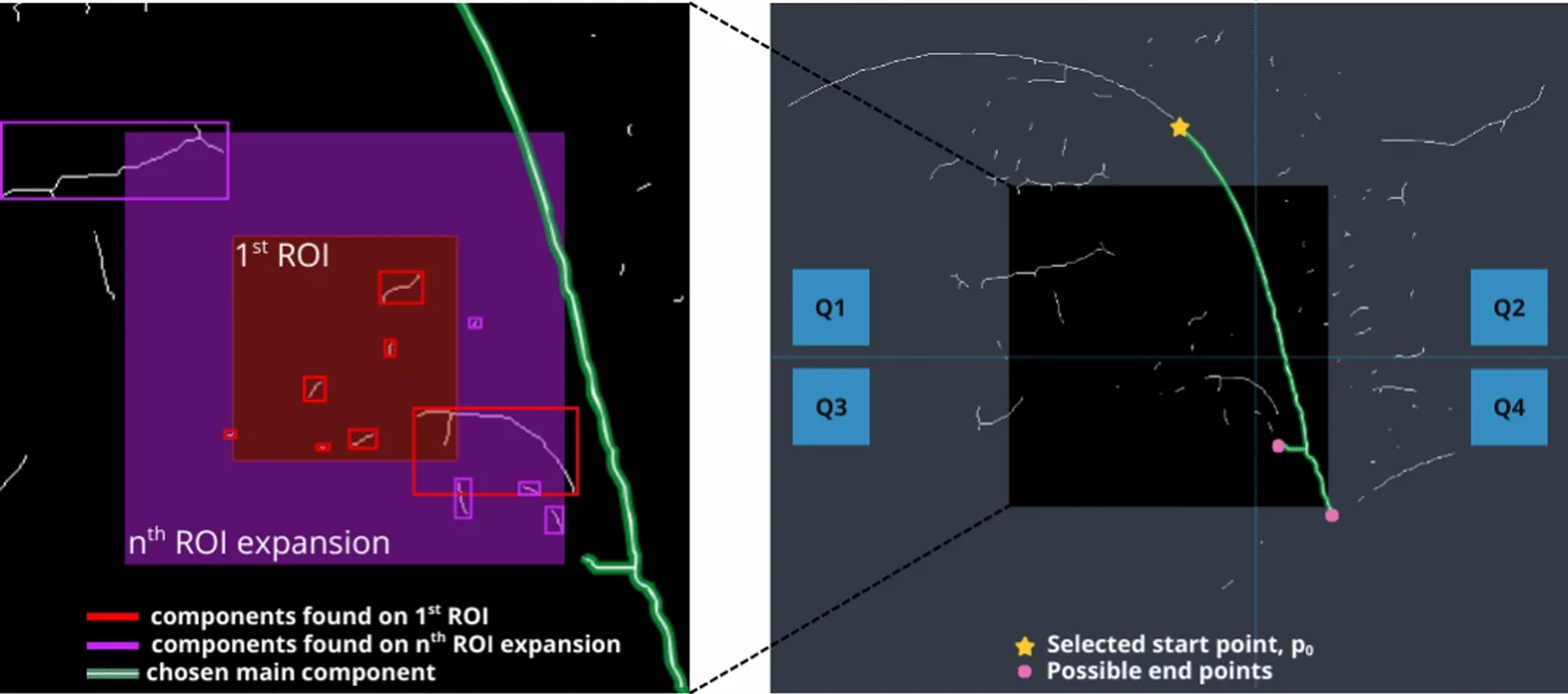
Main component selection algorithm. If components within the initial region of interest (ROI, red bounding boxes) do not meet the criteria, the ROI is iteratively expanded *n* times. On the $n^{th}$ expansion, components still not meeting the criteria are disregarded (purple bounding boxes). The main component (green) is selected as the largest component that satisfies the criteria. Its start point, $\textbf{p}_0$, is determined according to the quadrant-based rule set of Table 1

The main component forms the foundation for all subsequent components, making its accurate identification critical. In most swallow videos, part of the manometry catheter passes near the center of the frame. Leveraging this, our method first scans a smaller region-of-interest (ROI) centered in the frame, maintaining the frame’s aspect ratio. Within this ROI, we evaluate whether any connected component is $M_{min}$ the size of the largest component in the entire image. If none meets this criterion, the region is progressively expanded until a suitable candidate is identified (see Fig. 3).

To begin the path-growing analysis, the start point of the component, $\textbf{p}_0 = (x_0, y_0)$, is automatically selected from its edge points (EPs), using a hierarchical rule set based on the catheter’s spatial heuristics (cf. Table 1). The quadrant’s midpoint is defined based on the main component’s width, and the frame’s height (cf. Fig. 3).

**Table 1:** Catheter’s spatial heuristics rule set for determining the main component’s start point, $\textbf{p}_0$

Priority	Selection rule
1	Leftmost EP in Q1
2	Topmost EP in Q2 (if Q1 empty and Q2, Q4 populated)
3	Rightmost EP in Q4
4	Leftmost EP in Q3
5	Topmost EP in Q2 (if only Q2 populated)

Abbreviations: EP = edge point; Q*x* = quadrant *x*

#### Component pruning

As illustrated in Fig. 3, skeletonized components often exhibit branches originating from background complexity or swallowing dynamics. To ensure accurate catheter segmentation, we propose a path-growing pruning algorithm that traces smooth paths by recursively selecting points based on local neighborhood geometry and heading.

**Fig. 4:**
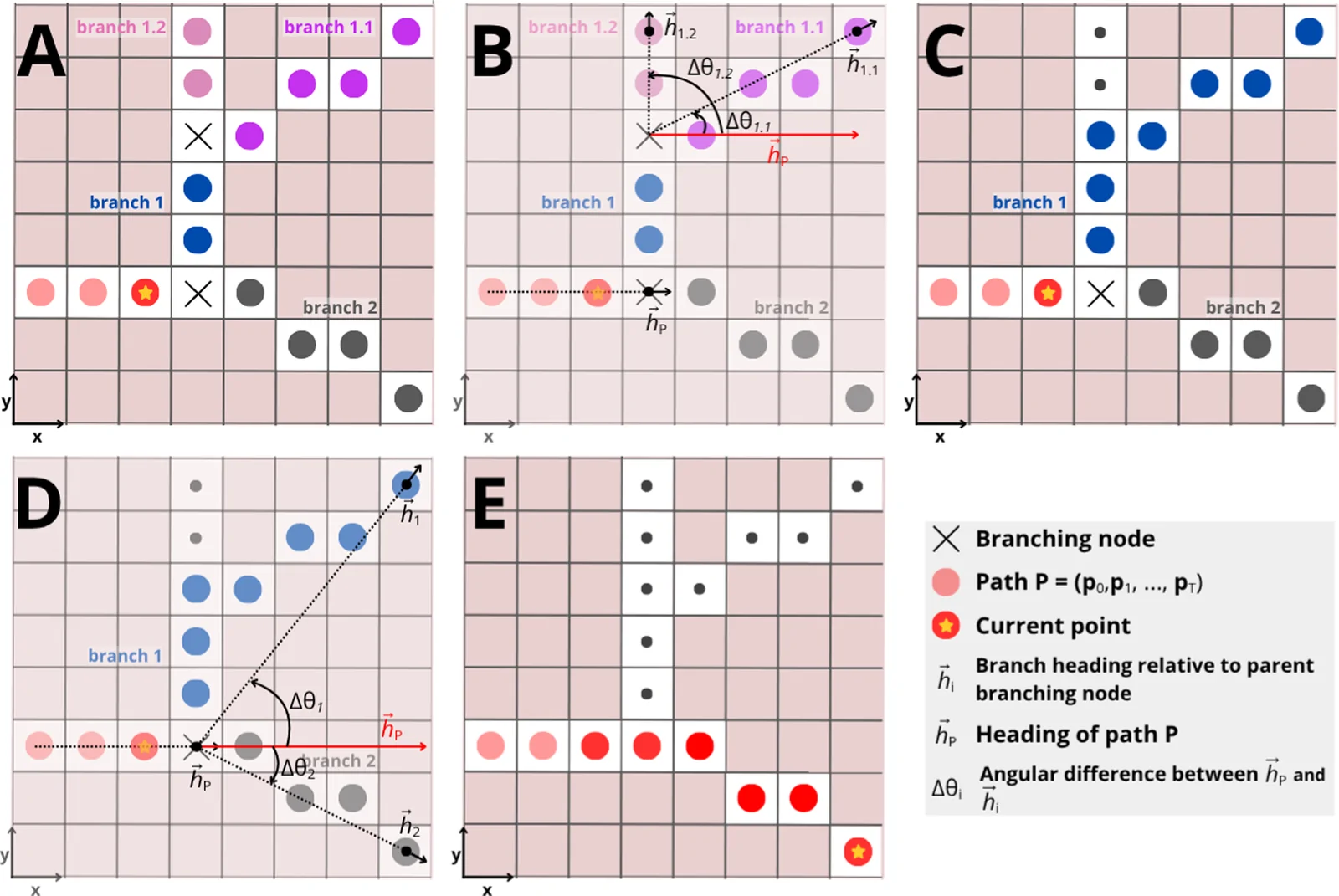
Pruning algorithm. In **A**, the current path $\textbf{P}$ reaches a branching node. The analysis begins with *branch 1*, where a sub-branching node is encountered. In **B**, each sub-branch of *branch 1* is explored for a maximum of $N_{steps}$. The heading of each sub-path, $\vec {h}_i$, is computed, and the absolute angular difference $|\Delta \theta _i|$ relative to the original path’s heading $\vec {h}_P$ is used to identify the sub-path that minimizes directional deviation. Based on this criterion, *branch 1.1* is selected as the most appropriate extension of *branch 1* (cf. **C**). After resolving the sub-branches, the first branching node is evaluated using the same approach (cf. **D**), and *branch 2* is ultimately chosen as the optimal extension of $\textbf{P}$

The path $\textbf{P} = (\textbf{p}_0, \textbf{p}_1, \dots , \textbf{p}_T), \quad \textbf{p}_t \in \mathbb {Z}^2,$ begins at the component’s start point, $\textbf{p}_0$. At each step, the current point $\textbf{p}_t$ selects the nearest unvisited neighbor from its 8-connected neighborhood, $\mathcal {N}(\textbf{p}_t) \subset \mathbb {Z}^2$, as the next point $\textbf{p}_{t+1} \in \mathcal {N}(\textbf{p}_t) {\setminus } \textbf{P}$. When $\textbf{p}_t$ has multiple options, each neighbor is treated as the root of a candidate branch. The pruning algorithm is then applied independently to each branch for a maximum of $N_{head}$ steps, ensuring correct handling of nested branches. This results in a set of sub-paths $\{ \Gamma _i \}_{i=1}^{k}$, each with an heading vector $\vec {h}_i$. To enforce directional continuity, the branch with the smallest absolute angular deviation $|\Delta \theta _i| = \angle (\vec {h}_{\textbf{P}}, \vec {h}_i)$ relative to the current path’s heading, $\vec {h}_{\textbf{P}}$, is selected, promoting smooth transitions and penalizing sharp turns. The process is applied recursively until an end EP of the component has been reached. A complete illustration of the methodology can be found in Fig. 4.

#### Component propagation

To complete the catheter segmentation with additional components, we propose a recursive directional adaptive-neighborhood search starting from the pruned main component.

A fixed-size ROI is propagated from each EP of the main component, hereafter referred to as the *parent component* and its corresponding *parent EP*. The heading direction of a parent EP, $\vec {h}_{\textit{EPi}}$, is defined as a unit vector rooted at the EP and oriented opposite to the incoming path $\textbf{P}$. When one or more components larger than $S_{\text {min}}$ fall within the ROI, they undergo the pruning procedure described in Sect. 2.4.2. In such cases, $\mathbf {p_0}$ is defined as the EP closest to the parent EP. Each candidate component is then assigned a score, $\gamma _c$, based on three measures:

1. the Euclidean distance from $\mathbf {p_0}$ to the parent EP, defined as $d_{0} = \Vert \mathbf {p_0} - \textbf{EP}_{\text {parent}} \Vert$;
2. the absolute angular deviation from the parent component’s heading given by $\Delta \theta _0 = \angle (\vec {h}_{EPi}, \vec {h}_0)$, where $\vec {h}_0$ denotes the heading direction of the candidate component;
3. the number of points in the component’s path $\textbf{P}$, given by $S_c$.

A custom scoring function integrates these metrics with weights α and β, balancing spatial and directional consistency. If a component’s angular deviation exceeds $\Delta \theta _{\text {max}}$, its score is set to zero, and the search continues for eligible components (see Eq. 1).

1 $$\begin{aligned} \gamma _c = {\left\{ \begin{array}{ll} \displaystyle \frac{S_{c}}{1 + \alpha d_{0} + \beta d_{0} \Delta \theta _0}, &  \text {if } \Delta \theta _0 \le \Delta \theta _{\text {max}} \\ 0, &  \text {otherwise} \end{array}\right. } \end{aligned}$$

**Fig. 5:**
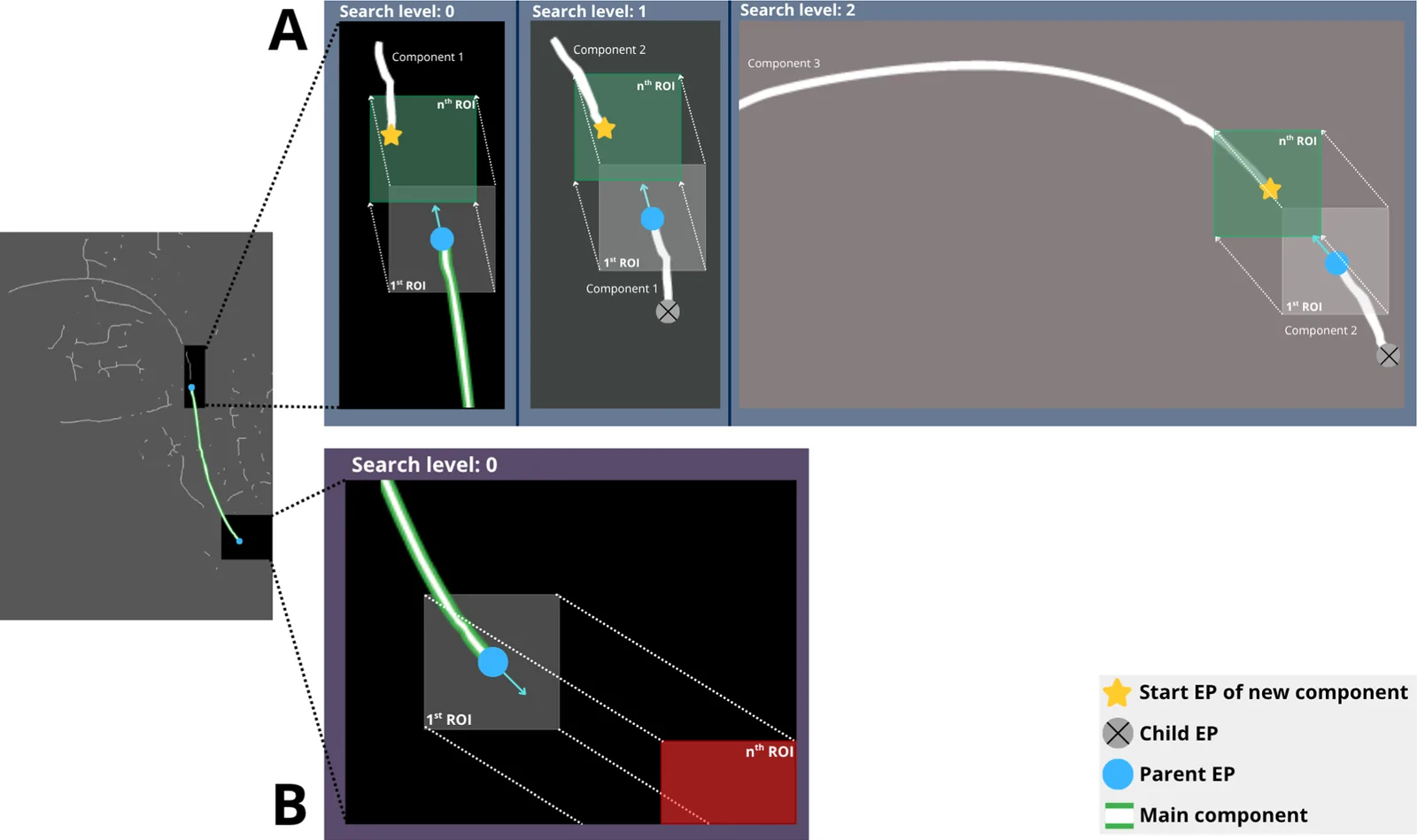
Component propagation algorithm. In **A**, the algorithm recursively searches for new components until none are found. When a new component is detected, its start edge point (EP) is set as the one closest to the parent EP. This start EP is marked as a child EP, and no further search is performed from it. In **B**, the search fails to find any new components before reaching the image boundary, at which point the search is terminated

The highest scoring component is added to the predicted catheter segmentation, and the search from its remaining EP (cf. Fig. 5). This process repeats recursively until no component exceeds the minimum score $\Gamma _{min}$ or the image boundaries are reached. All selected components collectively represent the predicted catheter segmentation.

### Hyperparameters

Table 2 summarizes the final hyperparameters used in our method, along with their descriptions. For this specific application, each parameter was empirically fine-tuned.

**Table 2:** Summary of empirically fine-tuned hyperparameters

Hyperparameter	Value	Section	Description
$M_{min}$	0.3	Sect. 2.4.1	Minimum relative size required for a connected component to be considered a valid main component candidate
$N_{head}$	40 steps	Sect. 2.4.2	Maximum number of steps used to estimate the heading of each candidate branch during pruning
$S_{min}$	5 px	Sect. 2.4.3	Minimum length a component must possess to be considered a valid extension of the main component
$\Delta \theta _{max}$	55 deg	Sect. 2.4.3	Maximum absolute angular deviation between the candidate component’s heading and the parent component’s heading to be considered a valid extension of the parent component
α	0.5	Sect. 2.4.3	Weight controlling the influence of Euclidean distance on the scoring function
β	0.5	Sect. 2.4.3	Weight controlling the influence of angular deviation on the scoring function
$\Gamma _{min}$	0	Sect. 2.4.3	Minimum score a component must achieve in order to be accepted as an extension of the main component

The Section column indicates where each hyperparameter is first introduced, and the Description summarizes its role in the algorithm

### Evaluation

The proposed segmentation methodology was first evaluated on the *Random Frames Dataset*, which isolates individual frames from all available swallow videos to test generalization. To evaluate the influence of temporal continuity, the algorithm was also tested on the *Full Videos Dataset*.

Performance was quantified using pixel-wise metrics *Precision* (P), *Recall* (R), and *F1-score* (F1), as defined in Eq. 2. Both the GT skeleton and the GT mask were used. *False Positives* (FP) were predicted pixels outside the GT mask. For each GT skeleton pixel, the orthogonal line to the local direction, denoted as $\perp \vec {h}_{GTi}$, was computed to classify *False Negatives* (FN) and *True Positives* (TP). A TP was counted when a predicted pixel was found along $\perp \vec {h}_{GTi}$ within the GT mask, otherwise the pixel was a FN.

2 $$\begin{aligned} \begin{aligned} P&= \frac{TP}{TP + FP} \\ R&= \frac{TP}{TP + FN} \\ F_1&= 2 \cdot \frac{P \cdot R}{P + R} \end{aligned} \end{aligned}$$

To compare with similar works, a distance threshold was inferred from the GT mask. This threshold was defined as half average length of $\perp \vec {h}_{GTi}$ along the GT skeleton, representing the maximum allowable deviation from the GT centreline for a predicted pixel to be considered a TP. The estimated average catheter width across all swallow videos was 3.54±0.28 mm, as such, the aforementioned distance threshold was 1.77±0.14 mm.

For each TP pixel, the Euclidean distance to its corresponding GT pixel, $d_{TP} = \Vert \mathbf {p_{TPi}} - \mathbf {p_{GTi}} \Vert$, was computed and converted to millimetres using the calculated scales. Additionally, the distance between each predicted pixels (TP and FP), $\mathbf {p_{i}}$, and its closest GT pixel, $d_{all} = \Vert \mathbf {p_{i}} - \mathbf {p_{GTi}} \Vert$, was calculated.

## Results

**Fig. 6:**
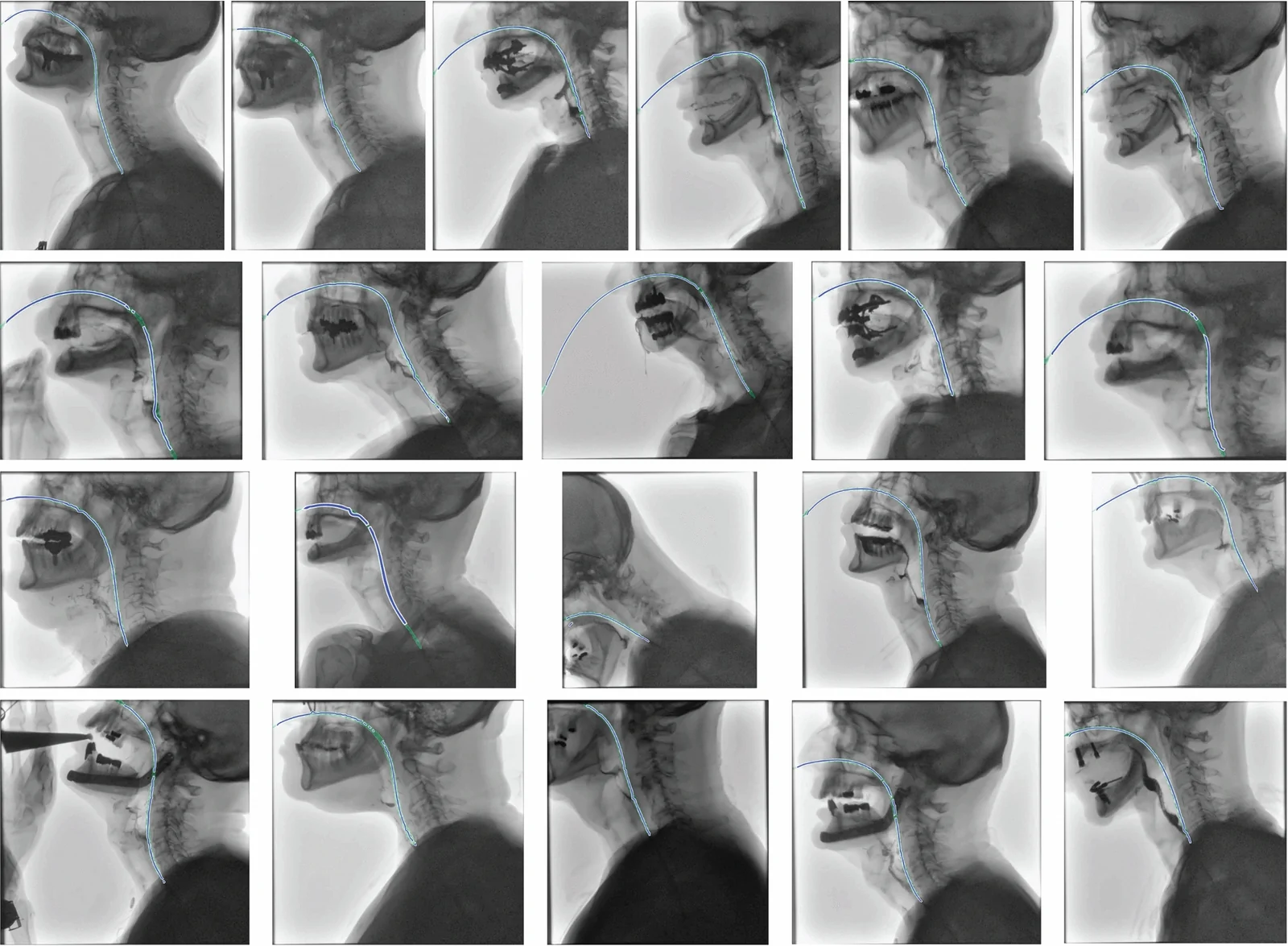
Catheter predictions (blue and white) of the base framework on the *Random Frames Dataset*. The predictions, originally skeletons, were enlarged for visualization purposes. The ground-truth mask is shown in green behind the predictions

Examples of representative predictions on the *Random Frames Dataset* are shown in Fig. 6. These examples illustrate the dataset’s variability, with the catheter appearing in different positions and lengths, often alongside bolus material, hands, or other structures, as well as variations in contrast and blurriness.

Table 3 reports the overall pixel-wise metrics and average distances to the GT centreline for the 976 images of the *Random Frames Dataset* and the 1631 frames of the *Full Videos Dataset*. On the *Random Frames Dataset*, the framework achieved 94% *Precision*, 84% *Recall*, and 88.5% *F1-score*, demonstrating strong generalization. On the *Full Videos Dataset*, performance improved to 95% *Precision*, 89% *Recall* and 92% F1-score, showing effective catheter tracking throughout complete swallow events. The estimated average catheter width across all videos was slightly higher than the actual 3.30 mm width.. This discrepancy reflects minor inaccuracies in the scaling factors. As a result, the reported distance errors may be negatively impacted (higher errors). Nonetheless, we can infer that FP predictions were generally far from the GT positions, as reflected by the substantial difference between $d_{all}$ and $d_{\textit{TP}}$.

**Table 3:** Summary of pixel-wise metrics of the segmentation framework, evaluated on the *Random Frames Dataset* and the *Full Videos Dataset*. The overall pixel-wise *Precision*, *Recall* and *F1-score* are presented, along with the overall $d_{TP}$ and $d_{all}$

Dataset	Precision	Recall	F1-score	${d_{TP}}$ (mm)	${d_{all}}$ (mm)
**Random Frames**	**0.938**	**0.838**	**0.885**	$\mathbf {0.26 \pm 0.01}$	$\mathbf {1.35 \pm 4.80 }$
**Full Videos**	**0**.**947**	**0**.**885**	**0**.**915**	$\mathbf {0.23\pm 0.10}$	$\mathbf {1.41 \pm 4.56}$
*Full Video 1*	0.864	0.722	0.786	0.32±0.17	4.14±8.16
*Full Video 2*	0.967	0.951	0.959	0.23±0.06	1.01±4.02
*Full Video 3*	0.956	0.915	0.935	0.24±0.04	0.52±1.91
*Full Video 4*	0.962	0.883	0.921	0.29±0.17	1.96±7.01
*Full Video 5*	0.967	0.939	0.953	0.18±0.02	0.32±0.46
*Full Video 6*	0.924	0.890	0.907	0.23±0.06	2.67±4.67
*Full Video 7*	0.978	0.801	0.881	0.17±0.05	0.25±0.40

**Fig. 7:**
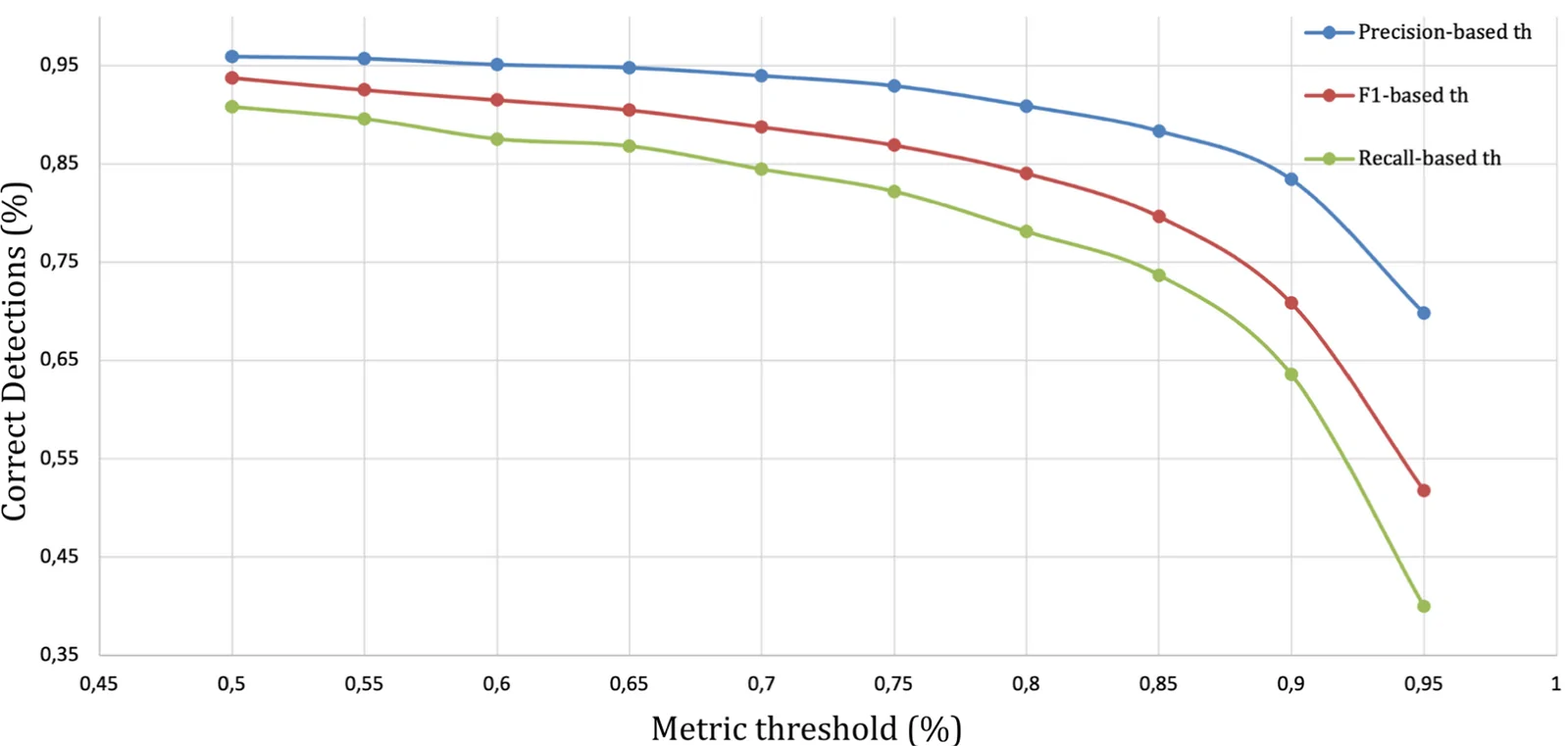
Variation of correct catheter detections on the *Random Frames Dataset* with different metric-based thresholds, namely pixel-wise *Precision*, *Recall* and *F1-score*

Figure 7 presents the percentage of correct predictions across varying detection thresholds using different metrics. The *Precision*-based curve remains above 90% up to a threshold of 0.85, demonstrating robustness to background disturbances. With a *Recall*-based threshold, the correct prediction rate drops below 80% beyond a 0.8 threshold. Nevertheless, using an *F1*-based threshold, the framework achieves nearly 85% correct predictions at a cut-off of 0.8.

## Discussion

In this study, we proposed a knowledge-based catheter segmentation algorithm designed to support sensor-based registration of HRIM and VFSS, thereby facilitating manometric region delineation in VFSS images. The framework integrates prior information on catheter shape and continuity to improve detection in dynamic VFSS environments. The algorithm performed robustly across swallow video frames from HNC patients, despite variability in anatomy, foreign objects, and partial obstructions. FP and FN rates are reported as complements of *Precision* and *Recall* for comparison with existing literature.

Despite differences in application domains, our segmentation algorithm consistently outperformed the approaches proposed by Hoffmann et al. [32], Omisore et al. [33] and [29]. On the *Random Frames Dataset*, our approach achieved 6.2% FP rate, and 16.2% FN rate, at a distance threshold of 1.77±0.14 mm. On the *Full Videos Dataset*, the framework achieved an overall 5.3% FP rate and 11.5% FN rate for the same distance threshold.

In comparison, Hoffmann et al. [32] exhibited a FP rate of 16.6% and a FN rate of 24.5% using a threshold of 2 mm, with a pixel-wise error of $2.3 \pm 5.6~\text {mm}$, whereas our method achieved lower average distances (see Table 3), and detected catheters in 85% of the cases at a *Precision*-based threshold of 0.9. Relative to Omisore et al. [33], who reported 58% FP rate, 48% FN rate, and a mean pixel-wise error 1.94 mm, and Wu et al. [29], who reported FP rates of 9.2%, 12.9%, and 18.2%, and FN rates of 44%, 47%, and 29.2%, our method shows substantial improvements under stricter spatial tolerances. Our findings are consistent with Wang et al. [31], who reported FP and FN rates of 9.62% and 9.88%, respectively, on short, low-contrast sequences at a distance threshold of 3 pixels. Compared to Geiger et al. [35], who reported an overall detection *F1-score* of 86% using a 30-pixel radius and 68% with a 10-pixel radius around the sensor’s location, our framework demonstrates superior performance under stricter conditions.^1^

Despite the strong performance compared to similar studies in the literature, the proposed method still presents limitations. The correct selection of the main component’s start point, $\mathbf {p_0}$, is critical, as an incorrect choice can cause pruning errors and faulty path propagation. Additionally, the current spatial heuristics were empirically fine-tuned for left-to-right oriented catheters, limiting its generalization to other catheter orientations. This could be improved by dynamically evaluating all EPs as potential start-points, and selecting the most consistent path, removing reliance on a predefined $\mathbf {p_0}$. Furthermore, the resulting catheter centreline retains an irregular shape. Although B-spline fitting was attempted to obtain a smoother catheter representation, the results were suboptimal. Single-frame predictions are susceptible to deviations from the catheter centreline, particularly in dynamic regions with partial occlusions and intensity fluctuations, causing the spline fitting to misalign with the true position. An alternative could involve pixel-wise analysis across the gaps between disconnected segments to guide merging more accurately.

All hyperparameters were empirically fine-tuned for the current task, which may constrain performance and adaptation to other fluoroscopy scenarios. Incorporating an automated optimization strategy, such as Bayesian optimization, could facilitate extension of the method to other applications involving thin radiopaque objects (e.g., guidewires, vascular catheters, endotracheal tubes).

Moreover, the current dataset is restricted to oropharyngeal HRIM-VFSS examinations and a single catheter type. As a result, the algorithm’s performance cannot be directly validated for other anatomical regions, such as esophageal HRIM-VFSS, medical scenes (e.g., endovascular procedures), or types of thin radiopaque objects. Nonetheless, the method’s generalizability is primarily determined by the quality of the pre-processing stage. Given the path-propagation nature of the algorithm and its independence from pixel intensities, it could likely be extended to other tasks and structures, provided that the catheter candidate regions are reliably extracted and the hyperparameters are appropriately fine-tuned.

The current framework does not meet real-time requirements, with an average processing time of approximately 1 s per frame on a CPU without parallel processing. To enhance clinical utility, particularly for live or near–real-time simultaneous HRIM-VFSS analysis, future work should incorporate computational optimizations such as parallel processing or GPU-accelerated implementations.

While the present study is directly tailored to simultaneous HRIM–VFSS examinations, its benefits can be extended beyond this setting. In routine clinical practice, the two modalities are frequently acquired asynchronously due to logistical reasons, with patients frequently referred to external centers for VFSS. In the short term, the incorporation of the proposed algorithm into the spatial registration framework could enable accurate annotation of large HRIM datasets using VFSS as visual guidance. In the longer term, once sufficient annotated data are available, these datasets can support the training of learning-based models for automated HRIM analysis, removing the need for VFSS feedback during routine use.

## Conclusion

We presented a template-free, knowledge-based framework for HRIM catheter segmentation in VFSS videos, designed to handle challenging imaging conditions, including occlusions, rapid motion, and anatomical variability. By leveraging geometric and directional priors, the method achieved accurate and interpretable catheter localization without relying on large annotated datasets, demonstrating robust generalization across diverse anatomies and imaging conditions.

The proposed framework represents an initial step toward computer-aided analysis of HRIM and VFSS. By automatically localizing the HRIM catheter in VFSS frames, the method lays the groundwork for subsequent sensor-level identification, enabling manometric region delineation directly on the imaging data. This approach is particularly valuable for populations with altered anatomy, such as HNC patients, where the manual delineation of manometric regions on HRIM plots is challenging and prone to errors.

Beyond HRIM–VFSS applications, the algorithm has the potential to generalize to other clinical scenarios requiring localization of thin radiopaque structures. Its independence from pixel intensities enables consistent frame-to-frame predictions, provided that candidate regions are reliably extracted and hyperparameters are properly tuned. Combined with its lightweight and transparent design, this makes the method a practical and versatile tool for clinical analysis.

## Footnotes

1 Our evaluation threshold of 1.77 mm corresponds to approximately 7 pixels.
